# Two-Level Domain Adaptation Neural Network for EEG-Based Emotion Recognition

**DOI:** 10.3389/fnhum.2020.605246

**Published:** 2021-01-20

**Authors:** Guangcheng Bao, Ning Zhuang, Li Tong, Bin Yan, Jun Shu, Linyuan Wang, Ying Zeng, Zhichong Shen

**Affiliations:** ^1^Henan Key Laboratory of Imaging and Intelligent Processing, PLA Strategic Support Force Information Engineering University, Zhengzhou, China; ^2^Key Laboratory for NeuroInformation of Ministry of Education, School of Life Science and Technology, University of Electronic Science and Technology of China, Chengdu, China

**Keywords:** EEG, emotion recognition, topological graph feature, maximum mean discrepancy, domain adversarial network

## Abstract

Emotion recognition plays an important part in human-computer interaction (HCI). Currently, the main challenge in electroencephalogram (EEG)-based emotion recognition is the non-stationarity of EEG signals, which causes performance of the trained model decreasing over time. In this paper, we propose a two-level domain adaptation neural network (TDANN) to construct a transfer model for EEG-based emotion recognition. Specifically, deep features from the topological graph, which preserve topological information from EEG signals, are extracted using a deep neural network. These features are then passed through TDANN for two-level domain confusion. The first level uses the maximum mean discrepancy (MMD) to reduce the distribution discrepancy of deep features between source domain and target domain, and the second uses the domain adversarial neural network (DANN) to force the deep features closer to their corresponding class centers. We evaluated the domain-transfer performance of the model on both our self-built data set and the public data set SEED. In the cross-day transfer experiment, the ability to accurately discriminate joy from other emotions was high: sadness (84%), anger (87.04%), and fear (85.32%) on the self-built data set. The accuracy reached 74.93% on the SEED data set. In the cross-subject transfer experiment, the ability to accurately discriminate joy from other emotions was equally high: sadness (83.79%), anger (84.13%), and fear (81.72%) on the self-built data set. The average accuracy reached 87.9% on the SEED data set, which was higher than WGAN-DA. The experimental results demonstrate that the proposed TDANN can effectively handle the domain transfer problem in EEG-based emotion recognition.

## Introduction

Emotion recognition plays an important role in the human-computer interaction system (Walter et al., 2014). In addition, accurately identifying the patient's emotions helps improve the quality of medical care (Acharya et al., 2015). Currently, popular emotion detection can be divided into two categories. One is based on non-physiological signals such as facial expressions (Gur et al., 1992). The other is based on physiological signals such as electroencephalogram (EEG) signals (Sourina et al., 2012). Facial expressions are prone to misinterpretation (Saxen et al., 2017), but EEG signals are directly extracted from the cerebral cortex without damage, accurately reflecting the physiological state of the human brain. Therefore, emotion recognition technology based on EEG signals has received more extensive research interest.

At present, researchers use a variety of traditional machine learning methods to identify emotions via EEG, including support vector machines (SVM) (Alarcao and Fonseca, 1949), linear discriminant analysis (LDA) (Zong et al., 2016), *K*-nearest neighbor (KNN) (Mehmood and Lee, 2015), and more. Although these methods have achieved good performance in EEG emotion recognition, there are still limitations. Due to the individual differences and non-stationarity of EEG signals, traditional machine learning methods have high requirements for extracted features. However, most of the current methods for extracting features from EEG signals are manual, and the results are often not satisfactory.

Researchers have proposed a variety of shallow unsupervised domain adaptation methods to solve the cross-subject classification problem. The main idea of this shallow unsupervised domain adaptation method is to learn shared features by minimizing the distance of the distribution difference between features from different domains. Algorithms for measuring the distance between two distributions usually include KL divergence, Wasserstein distance, Shannon entropy distance, and maximum mean discrepancy (MMD) (Chai et al., 2016). In recent years, the multiple kernel maximum mean discrepancy (MK-MMD) (Hang et al., 2019) has shown a greater advantage in domain adaptation. Pan et al. (2011) proposed a domain adaptation method called Transfer Component Analysis (TCA). The principle was to map two differently distributed data points to a high-dimensional regenerative kernel Hilbert space (RKHS) by learning a set of universal transfer mappings between the source and target domains, and then minimize the MMD in the RKHS to minimize the distribution distance between the source and target domains. The Transformation Parameter Transfer (TPT) method proposed by Sangineto et al. (2014) first trained the classifier of each source domain, then trained a regression function to learn the relationship between the data distribution and the classifier parameters, and finally used the target domain distribution and classifier mapping to obtain the target classifier, thereby realizing distribution transfer. The shallow domain adaptation method has achieved remarkable results in cross-subject classification, but its performance depends in large part on the quality of the features and the classification performance of the classifier. However, it is well-known that it is very difficult to design a general classifier. If the extracted features are inaccurate, the resulting model may lead to reduced classification performance, that is, negative transfer.

Therefore, researchers are more interested in deep domain adaptation methods. Studies have found that deep neural networks can learn more transferable features for domain adaptation (Donahue et al., 2013; Yosinski et al., 2014). Ganin et al. (2016) proposed a domain-adversarial training of neural networks (DANN), an approach composed of two main parts. First, the source and target domains were mapped to a common subspace through shared parameters for alignment, and then the source domain classification loss was minimized. Domain classification loss of the source and target domains was maximized to achieve domain confusion. The deep adaptation network (DAN) (Hang et al., 2019) proposed by Long et al. relied on multi-kernel MMD (MK-MMD) to adapt the source domain and target domain after multiple fully connected layers in the deep layer. In addition, Luo et al. (2018) proposed a domain adaptation framework based on WGAN. There were two main steps; the first was to pre-train the source domain, and then the Wasserstein algorithm was used for adversarial training to adapt the target domain to the source domain. Similar to the WGAN framework, Jimenez-Guarneros and Gomez-Gil (2020) proposed a custom domain adaptive method (CDA). This method used adaptive batch normalization (AdaBN) (Li et al., 2018) and MMD in two independent networks to reduce the marginal and conditional distribution of the source and target domains. Ma et al. (2019) proposed an adversarial domain generalization framework called DResNet, which learned specific biased weights for each source domain and unbiased weights shared by all domains. Unlike the other methods mentioned above, this method did not use any information about the target domain. At present, most of the methods based on deep domain adaptation put the distributed adaptation strategy on the specific task layer of the deep network, which can better reduce the domain difference. However, these deep domain adaptation methods usually only use simple distributed adaptation methods, which cannot confuse the source domain and target domain well. In addition, most of the existing deep domain adaptation methods are based on image classification, and there are few domain adaptation methods based on cross-subject EEG emotion classification. For example, Zheng and Lu (2016) proposed a framework of emotion transfer based on TPT, Luo et al. (2018) proposed a domain adaptation method for EEG emotion based on WGAN, Li Y. et al. (2019) proposed a domain adversarial method for EEG emotion based on Bi-hemisphere, Li J. et al. (2019) proposed a multisource transfer method for EEG emotion, Li et al. (2020) proposed a domain adaptation method for EEG emotion based on latent representation similarity.

Clearly, even if a subject induces the same emotion at different times, some external factors such as temperature and humidity will cause physiological changes (Chueh et al., 2012). This will cause changes in their EEG signals that are called cross-day variability. At present, few researchers analyze and study this problem. Although the tasks of cross-day transfer and cross-subject transfer are the same, they both match the distribution of source domain and target domain to eliminate the distribution difference. But they have different characteristics to learn. The challenge in cross-day transfer is to train a general classification model for the same subject, which must extract the same EEG features for the same emotional states across days. Cross-subject transfer, on the other hand, trains a general classification model for different subjects, and must extract the same EEG features for the same emotional states across subjects. It is very difficult to build a general model and extract high-quality features; a deep neural network is better than traditional methods at learning features.

In this paper, we propose a two-level deep domain adversarial network model based on a deep convolutional neural network to recognize EEG emotion transfer. EEG features are mapped to images, and the spatial topological information of EEG features is simultaneously retained using the method presented by Bashivan et al. (2015) and Hwang et al. (2020). A deep convolutional neural network can learn more transferable features by learning the EEG feature topological map. We use the AdaBN layer to standardize the characteristics of the source and target domains, and then use MMD to reduce the distribution difference between the source and target domains to achieve the domain matching effect. Finally, through the adversarial domain adaptation network, the distribution difference between the source and target domains is further reduced dynamically to achieve complete domain confusion. We verified the cross-day transfer and cross-subject transfer.

The main contributions of this manuscript lie in the following aspects:

1. A two-level domain adaptation neural network (TDANN) was proposed to construct a transfer model for EEG-based emotion recognition. Through the combination of MMD and DANN, the source domain, and the target domain can adapt to each other better.
2. Topology features were used to increase spatial information, which can better describe the state of different emotions. In addition, a convolutional network with adaptive standard layer was proposed to extract effective emotion features from topology graph.
3. A cross-subject and cross-day emotion EEG data set was constructed to study the transfer models for EEG-based emotion recognition. In this data set, each subject participated in six sessions, which is the largest number of sessions in the current public datasets for EEG-based emotion recognition.

## Experimental Setup

Since there is no data set big enough for research on the cross-day transfer model for EEG-based emotion recognition, we designed an experiment to build an EEG data set for emotion recognition. Each subject's EEG signals under different emotion states were collected three times with a 1 week interval, and the sequence was repeated again after 1 month.

### Stimuli and Experimental Procedure

Thirty-six video clips of joy, sadness, anger, and fear were chosen for the experiment from the Chinese affective video system (Xu et al., 2010) and from a self-built emotional material library. The self-built library was a standardized multi-sensory emotional stimulation material library built on the basis of psychological methods and composed of various comedy, love, crime, war, documentary, and horror films with a clear picture and good sound. In order to induce a single type of emotion accurately, the length of movie clips was set to 50–335 s and the emotion induced by each video reached the highest intensity at the end.

**Figure 1:**
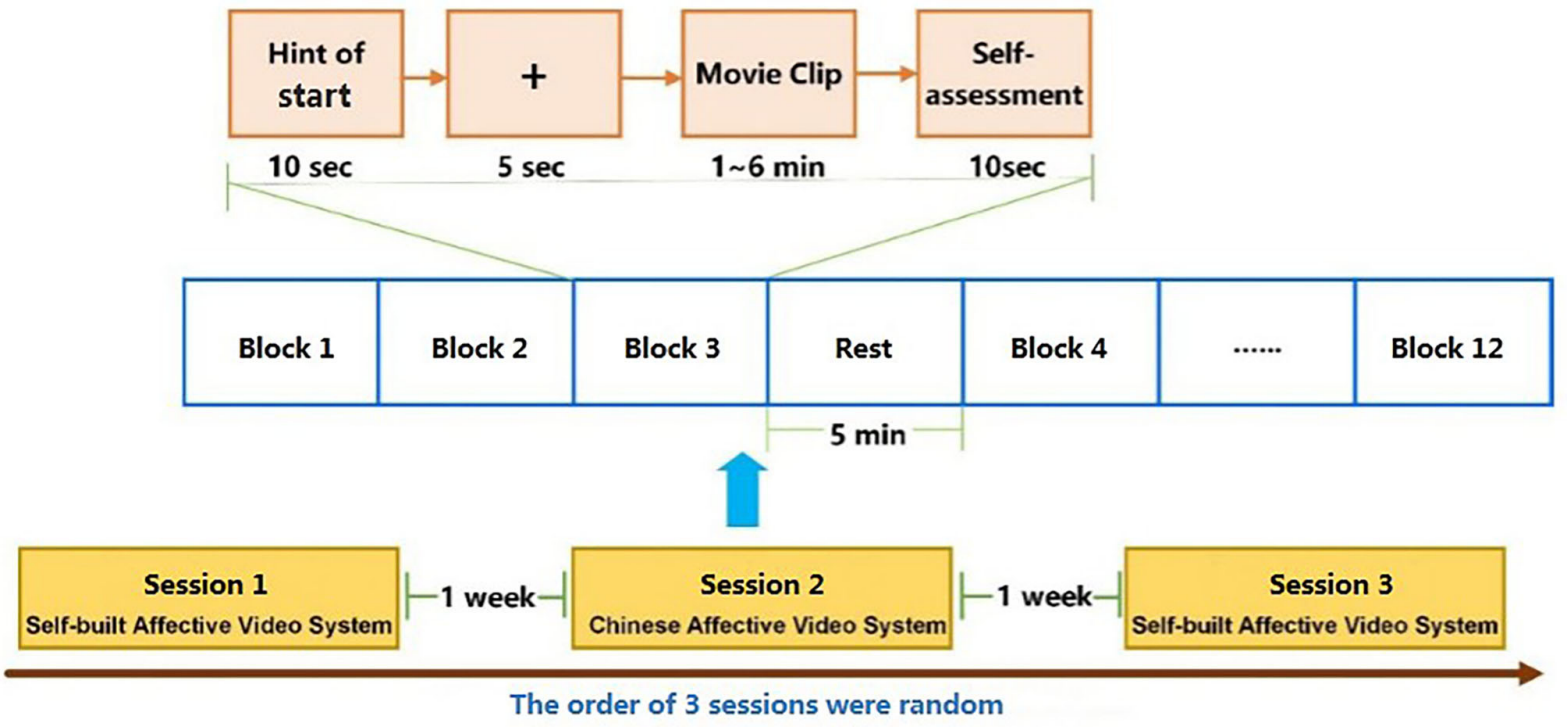
Experimental procedure. The experiment was performed in three parts: Experiments A, B, and C. The order of the three parts was random and the time interval was 1 week. In each part, 12 movie clips with four discrete categories of emotion (joy, sadness, anger, and fear) were presented in 12 trials. Each subject participated in two complete experiments.

The experiment was performed in three parts, namely, Experiments A, B, and C. The details of the movie clips used in each part are listed in Table 1. See Figure 1 for an overview of the experimental procedure.

**Table 1:** Brief description of the movie clips used in the emotion experiment.

**No**.	**Label**	**Experiment A**		**Experiment B**		**Experiment C**	
		**Movie Name**	**Length (sec)**	**Movie Name**	**Length(sec)**	**Movie Name**	**Length (sec)**
1	Joy	More Haste Less Speed	109	The Eagle Shooting Heroes (1)	228	Lost on Journey	281
2	Joy	A Big Potato	142	A World Without Thieves	191	Home with Kids	187
3	Joy	Flirting Scholar	112	Chaplin Comedy	244	The Eagle Shooting Heroes (2)	53
4	Sadness	My Brothers and Sisters	146	Dearest (1)	182	Man Phoning in the Snow	142
5	Sadness	Mother Love Me Once Again	137	Tangshan Earthquake;	335	Echoes of the Rainbow	241
6	Sadness	Warm Spring	102	Dearest (2)	120	ROB-B-HOOD	234
7	Anger	Fist of Fury (2)	66	YiP Man II	172	Japanese Aggression	96
8	Anger	Kangxi Dynasty	94	Don't Talk to Strangers	205	Blind Mountain	275
9	Anger	Conman in Tokyo	107	Fist of Fury (1)	258	Poaching Wild Animals	148
10	Fear	Help Me	50	Lights Out	134	A Man Lying in Bed	162
11	Fear	The Game of Killing (1)	159	Man Lying on the Ground	291	The Grudge	167
12	Fear	Inner Senses	247	Snake Eating People	158	A Woman Taking a Gun	190

The order of the three parts was random, and the time interval between them was 1 week. In each part, four categories of movie clips (total of 12 movie clips) were randomly presented to the participants in 12 trials, and each trial involved the following steps:

1. 10-s display of the current trial number to inform the participants of their progress
2. 5 s of baseline signal collection (fixation cross)
3. Display of the movie clips
4. 10-s self-assessment for arousal and valence (based on self-assessment manikins)
5. 5 min break between different emotional types of video clips.

### EEG Recording and Preprocessing

The Beck Anxiety Inventory (Fydrich et al., 1992), Hamilton Anxiety Rating Scale (Shear et al., 2010), and Hamilton Depression Scale (Hamilton, 2004) were administered to exclude individuals with anxiety, depression, or physical abnormalities and those under sedatives and psychotropic drugs. The participants included 16 college students (eight males and eight females) with an average age of 23.13 years (range = 19–27, SD = *r* 2.37). All participants were right-handed, with normal or corrected vision and hearing.

EEG signals were recorded with a gtec.HIamp system. The sampling rate was 512 Hz, a band-pass filter in the range of 0.1–100 Hz was utilized to filter EEG signals, and a notch filter with a frequency of 50 Hz was used. The layout of 62 electrodes followed the international 10–20 system. The Fz electrode was used for reference calculation. Thus, the number of effective electrodes was 61.

First, we selected the subjects' EEG data based on their self-evaluated valence. The threshold was set to 5. If a participant's valence for happy videos exceeded five points, and videos with sadness, anger, and fear were <5, we believed that the participant's emotions were accurately induced, and the participant's signal was retained; otherwise the participant's signal was deleted. We also excluded subjects with poor EEG signal quality, for example large EMG artifacts or EEG signal drift. In the end, we eliminated 4 subjects and retained 12 subjects with better signals. Then, we selected the last 50 s of the EEG signal from each video clip for analysis. In the video material, the shortest video length is 50 s. In order to make the sample balanced, we intercepted the data corresponding to all videos in the last 50 s. The EEG signals were passed through a 2-s time window and overlapped by 50%. After segmentation, each video segment had a total of 49 samples, and each participant had a total of 588 samples. There were 3,528 samples over 6 days.

Before extracting features, the data was preprocessed. First, the channels with poor data were recompressed and averaged with the surrounding channels. Next, the blind source analysis algorithm FastICA (Hyvärinen, 1999) was used to remove EOG artifacts. We used FastICA to decompose the original EEG signal into multiple ICs, identifying IC with occasional large amplitude as eye-movement artifact and removed it. Third, we used a band-pass filter of 0.1–64 Hz to filter out high-frequency interference in EEG signals. Then, we used the reference electrode standardization technology (REST) to re-reference the data (Yao, 2001; Yao et al., 2019), and finally, we removed the 5 s of the baseline before the task from the EEG signal.

## Two-Level Domain Adaptation Neural Network

**Figure 2:**
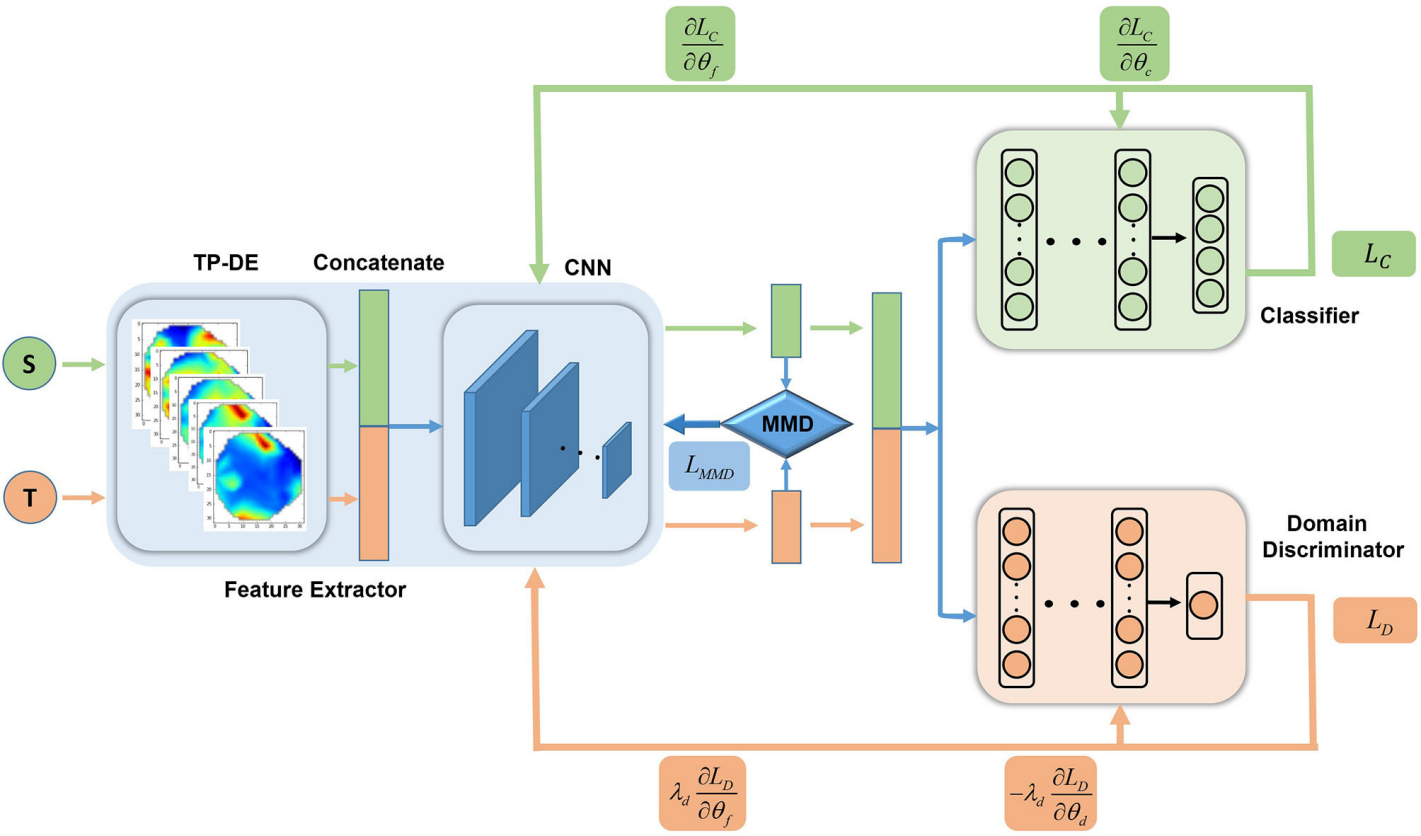
Flowchart of the two-level depth domain adaptation framework, with feature extractors, classifiers, and domain discriminators. The DE feature was converted into a topological map feature as the input of the feature extractor. After processing by the two-level domain adaptation network, the source and target domains were distributed similarly while ensuring classification performance. The first-level domain adaptation network was mainly composed of feature extractors and a traditional algorithm MMD; the second-level domain adaptation network was composed of domain adversarial networks with feature extractors and domain discriminators.

The two-level deep domain adaptation framework for EEG-based emotion recognition is shown in Figure 2. The framework was mainly composed of three parts, namely a feature generator, a domain discriminator, and a classifier. The main task of the generator was to further learn the stable features related to the emotional state in the EEG image and to align the source and target domains in the subspace. The domain discriminator further reduced the distribution distance between the source and target domains.

### Feature Generator Based on CNN

Feature extraction is a very critical step in the research of EEG emotion recognition. Features based on EEG emotion recognition are mainly divided into three categories: time-domain features, frequency-domain features, and time-frequency features (Jenke et al., 2014). Time domain features include energy, average, standard deviation, first-order variance, standard first-order variance, second-order variance, and standard second-order variance. Hjorth (1970) proposed more complex temporal characteristics: Activity, Mobility, and Complexity. There is also the fractal dimension (FD) (Sourina and Liu, 2011), in addition to the high-order cross (HOC) (Petrantonakis and Hadjileontiadis, 2010) feature extraction method, which represents the oscillation mode of the signal and has high stability. The frequency domain features are mainly extracted on five frequency bands, Delta band (1–3 Hz), Theta band (4–7 Hz), Alpha band (8–13 Hz), Beta band (14–30 Hz), and Gamma band (31–50 Hz). Commonly used frequency domain features include energy and power spectral density (PSD) (Jenke et al., 2014). Moreover, time-frequency domain features include differential entropy (DE) (Duan et al., 2013), differential asymmetry (DASM) feature, rational asymmetry (RASM) feature, and differential causality (DCAU) feature (Zheng et al., 2019). Time-frequency domain features is usually extracted by short-time Fourier transform (STFT) (Koenig, 1946), Hilbert-Huang Spectrum (HHS) (Hadjidimitriou and Hadjileontiadis, 2012), discrete wavelet transform (DWT) (Mallat, 2009) and other time-frequency transformation methods. Murugappan et al. (2010) used DWT to extract the energy and entropy of five frequency bands of EEG signal, including root mean square (RMS), and recursive energy efficiency (REE). Alazrai et al. (2018) proposed a quadratic time-frequency distribution (QTFD) to extract time-frequency feature. Most of the current researches extract the DE features of five frequency bands for emotion recognition. Since the EEG signal is non-stationary, it can be approximated that the EEG signals follow the Gaussian distribution *N*(μ, σ^2^), DE can be simply expressed by the following (Duan et al., 2013):

(1) $$\begin{array}{l} h(X)=-\int_{\infty }^{\infty }\frac{1}{\sqrt{2\pi \sigma^{2}}}\ell^{-\frac{{\left(x-\mu \right)}^{2}}{2\sigma^{2}}}\text{log }\left(\frac{1}{\sqrt{2\pi \sigma^{2}}}\ell^{-\frac{{\left(x-\mu \right)}^{2}}{2\sigma^{2}}}\right)dx\\ \;=\frac{1}{2}\text{log }\left(2\pi \ell \sigma^{2}\right) \end{array}$$

Where *X* submits the Gaussian distribution*N*(μ, σ^2^), $\frac{1}{\sqrt{2\pi \sigma^{2}}}\ell^{-\frac{{\left(x-\mu \right)}^{2}}{2\sigma^{2}}}$ is the probability density function of *X, x* is a variable, π and ℓ are constants.

**Figure 3:**
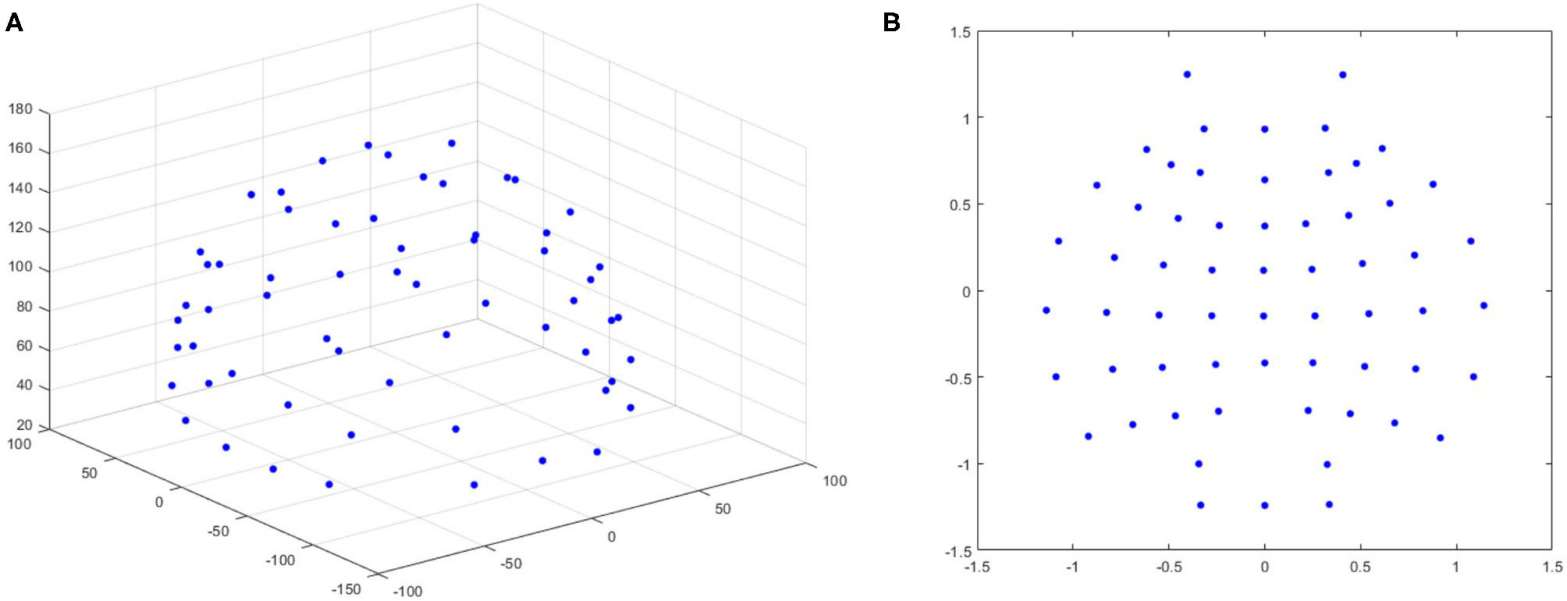
**(A)** 3-dimensional position of the EEG electrode with 61 channels. **(B)** 2-dimensional position of the electrode using the polar coordinate projection method.

**Figure 4:**
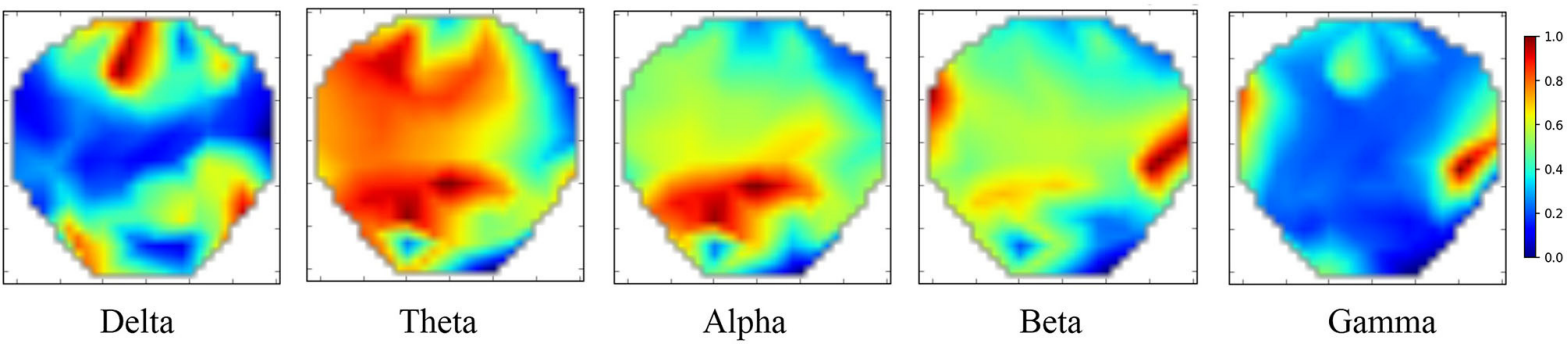
TP-DE images of five frequency bands for a certain participant. Length and width are 32; channel is 5.

The extracted DE features only consider the temporal information and ignore spatial information. Therefore, we adopted a previously tested method using polar coordinate projection to maintain the spatial topology (Bashivan et al., 2015; Hwang et al., 2020). We projected the three-dimensional electrode position onto a two-dimensional plane, as shown in Figure 3. We used the Clough–Tocher scheme interpolation method to insert the differential entropy feature on each electrode and to estimate the value between the electrodes to obtain a 32 × 32 × 5 EEG image. Figure 4 shows the topology-preserving DE (TP-DE) characteristics of five frequency bands of a certain subject after using maximum and minimum standardization.

In the deep CNN, we used a multi-layer convolutional layer and two maximum pooling layers. Table 2 shows the CNN model structure for cross-day transfer research. We added an AdaBN layer after each set of convolutional layer and fully connected layer. The AdaBN standardized the distribution between the source and target domains in each batch of samples, so that the source and target domains were better matched in the subspace. Each fully connected layer used a dropout layer, with dropout rate = 0.5.

**Table 2:** CNN model structure for cross-day transfer research.

**Layer**	**Input dimension**	**Output dimension**	**Kernel size**	**Stride size**
Conv1	5	6	3 × 3	1 × 1
Maxpool1	6	6	2 × 2	2 × 2
Conv2	6	64	3 × 3	1 × 1
Maxpool2	64	64	2 × 2	2 × 2
FC1	7 × 7 × 64	512		
FC2	512	256		

*In the mean, bolding means the accuracy mean is the largest; In the Std, the bold is the smallest*.

### Two-Level Domain Adaptation Method

In order to understand the deep domain adversarial method more clearly, we first introduce the symbols that will be used here. We assume $X_{S}\sim x_{i}^{S},i=1\cdots n_{S}$ is the data sample of the source domain*D*_*s*_, $Y_{S}\sim y_{i}^{S},i=1\cdots n_{S}$ is the label corresponding to the source domain data sample, and $X_{T}\sim x_{i}^{T},i=1\cdots n_{T}$ is the data sample of the target domain *D*_*T*_.

Feature generator *G*_*f*_maps the source domain data *X*_*S*_and the target domain data *X*_*T*_ to the same space:

(2) $$\begin{array}{r} X_{S}^{\prime }=G_{f}\left(X_{S}\right),X_{T}^{\prime }=G_{f}\left(X_{T}\right) \end{array}$$

The generator *G*_*f*_ shares parameters in the source domains *X*_*S*_ and target domains *X*_*T*_, so the feature dimensions of ${X^{\prime }}_{S}$ and ${X^{\prime }}_{T}$ are the same.

The function of the domain discriminator *G*_*d*_ is to distinguish the source domain and the target domain. It takes ${X^{\prime }}_{S}$ and ${X^{\prime }}_{T}$ as the input, and outputs the prediction of domain, respectively $Y_{S}^{D}$ and $Y_{T}^{D}$:

(3) $$\begin{array}{r} Y_{S}^{D}=G_{d}\left(X_{S}^{\prime }\right),Y_{T}^{D}=G_{d}\left(X_{T}^{\prime }\right) \end{array}$$

The role of the classifier *G*_*c*_ is to classify EEG emotions. It takes ${X^{\prime }}_{S}$ and ${X^{\prime }}_{T}$ as inputs and outputs predictive labels, which *Y*_*S*_ are and *Y*_*T*_:

(4) $$\begin{array}{r} Y_{S}=G_{c}\left(X_{S}^{\prime }\right),Y_{T}=G_{c}\left(X_{D}^{\prime }\right) \end{array}$$

We parameterize the generator *G*_*f*_, domain discriminator *G*_*d*_, and classifier *G*_*c*_; their parameters are θ_*f*_, θ_*d*_ and θ_*c*_ respectively.

First, we optimize the parameters and minimize the cross-entropy:

(5) $$\begin{array}{r} \underset{\theta_{f},\theta c}{\min}L_{C}\left(X_{S},X_{T}\right)=-{\text{E}}_{\left(x_{S},y_{S}\right)-\left(X_{S},Y_{S}\right)}\left[\overset{M}{\underset{c\;=\;1}{\sum }}y_{c}^{S}\log G_{c}\left(G_{f}\left(x_{S}\right)\right)\right] \end{array}$$

Here, *M* represents the emotion class.

Then, introducing the domain adaptation algorithm, we propose a two-level domain adaptation algorithm based on a deep neural network. In the first-level domain adaptation, we use the MMD algorithm, combined with the AdaBN layer in the feature extractor, to align the class distribution of the source and target domains. Under the premise of ensuring the classification performance, the source and target domains are initially confused, and the MMD distance is minimized by optimizing the parameter θ_*f*_:

(6) $$\underset{\theta_{f}}{\min}\;L_{MMD}(X_{S},X_{T})=L_{MMD}E_{X_{S},X_{T}}(X_{S},X_{T})$$

Where $L_{MMD}$ represents the MMD distance. MMD distance can effectively measure the distance between distributions, and can be expressed by:

(7) $$\begin{array}{rc} L_{MMD}\left(X_{S},X_{T}\right)=\frac{1}{n_{S}^{2}}\overset{n_{S}}{\underset{i,j\;=\;0}{\sum }}\kappa \left(X_{S}^{\left(i\right)},X_{S}^{\left(j\right)}\right)-\frac{1}{n_{S}n_{T}}\overset{n_{s},n_{T}}{\underset{i,j\;=\;0}{\sum }}\kappa \left(X_{S}^{\left(i\right)},X_{T}^{\left(j\right)}\right)\\ + & \frac{1}{n_{T}^{2}}\overset{n_{T}}{\underset{i,j\;=\;0}{\sum }}\kappa \left(X_{T}^{\left(i\right)},X_{T}^{\left(j\right)}\right) \end{array}$$

Where *n*_*S*_, *n*_*T*_ represent the number of samples in the source and target domains, respectively, and κ(·, ·) is a linear combination of multiple radial basis function (RBF) kernels, defined as:

(8) $$\begin{array}{r} \kappa \left(X_{S}^{\left(i\right)},X_{T}^{\left(j\right)}\right)=\underset{n}{\sum }\eta_{n}\exp\left\{-\frac{1}{2\sigma_{n}}{\left||X_{S}^{\left(i\right)}-X_{T}^{\left(j\right)}|\right|}^{2}\right\} \end{array}$$

Where σ_*n*_ is the standard deviation of the *n*^*th*^ RBF kernel and η_*n*_ corresponds to its associated weight.

Using the MMD algorithm alone for domain adaptation is not sufficient for multi-source domain matching. Therefore, the second-level domain adaptation–domain adversarial method is introduced. We use the second-level domain adaptation network to reduce the distribution distance between the source and target domains. The principle of the domain discriminator is to maximize the cross entropy by optimizing the parameters θ_*f*_ and θ_*d*_:

(9) $$\begin{array}{r} \underset{\theta_{d}}{\text{max}},\underset{\theta_{f}}{\min}L_{D}\left(X_{S},X_{T}\right)=-{\text{E}}_{\left(x_{S},x_{T}\right)-\left(X_{S},X_{T}\right)}\\ \left[\overset{N}{\underset{d\;=\;1}{\sum }}y_{d}^{}\log G_{d}\left(G_{f}\left(x_{S},x_{T}\right)\right)\right] \end{array}$$

Where *N* is the numbers of domains.

Finally, we add gradient penalty to the domain loss to realize the Lipschitz constraint, so that the domain loss function can be more stable and converge faster in training. We also add an extra *L*2 norm regular term:

(10) $$\begin{array}{r} \underset{\theta_{f},\theta_{c},\theta_{d}}{\min}L_{G}=L_{C}+\lambda_{d}L_{D}+\lambda_{m}L_{MMD}+\lambda_{z}{\left||W|\right|}_{2} \end{array}$$

(11) $$\begin{array}{r} \underset{\theta_{d}}{\max}L_{D}=-L_{D}+\lambda_{L}{\left({\left||\nabla_{x}G_{d}\left(x\right)|\right|}_{2}-1\right)}^{2} \end{array}$$

Where λ_*d*_, λ_*m*_, λ_*z*_, and λ_*L*_ are hyper-parameters, and is the transformation matrix.

## Results

### Cross-Day Transfer Research

We used a self-built data set for cross-day transfer research. In this data set, each participant had 6 days of data and each participant iterated six times. We used the leave-one-out method for cross-validation, that is, for each subject, 1 day was randomly selected as the test set, and the remaining days as the training set. In the deep network, 15% of the data was randomly selected from the training set every day as the validation set. In the parameter settings of the network model, the batch size was 160, the source and target domains were each 80, and the number of neurons in the fully connected layer was 512 and 256, respectively. The hyperparameters were λ_*d*_ (0.1), λ_*m*_ (0.1), λ_*z*_ (0.01), and λ_*L*_ (10). An Adam optimizer was used, and the learning rate was 0.0005. All the methods in this paper were implemented in Python, and the deep neural network was implemented in Tensorflow. The workstation operating system was Windows 7, using Inter(R)Xeon(R) E3-1230v3 CPU, NVIDIA TITAN V GPU, and 16G of RAM.

**Figure 5:**
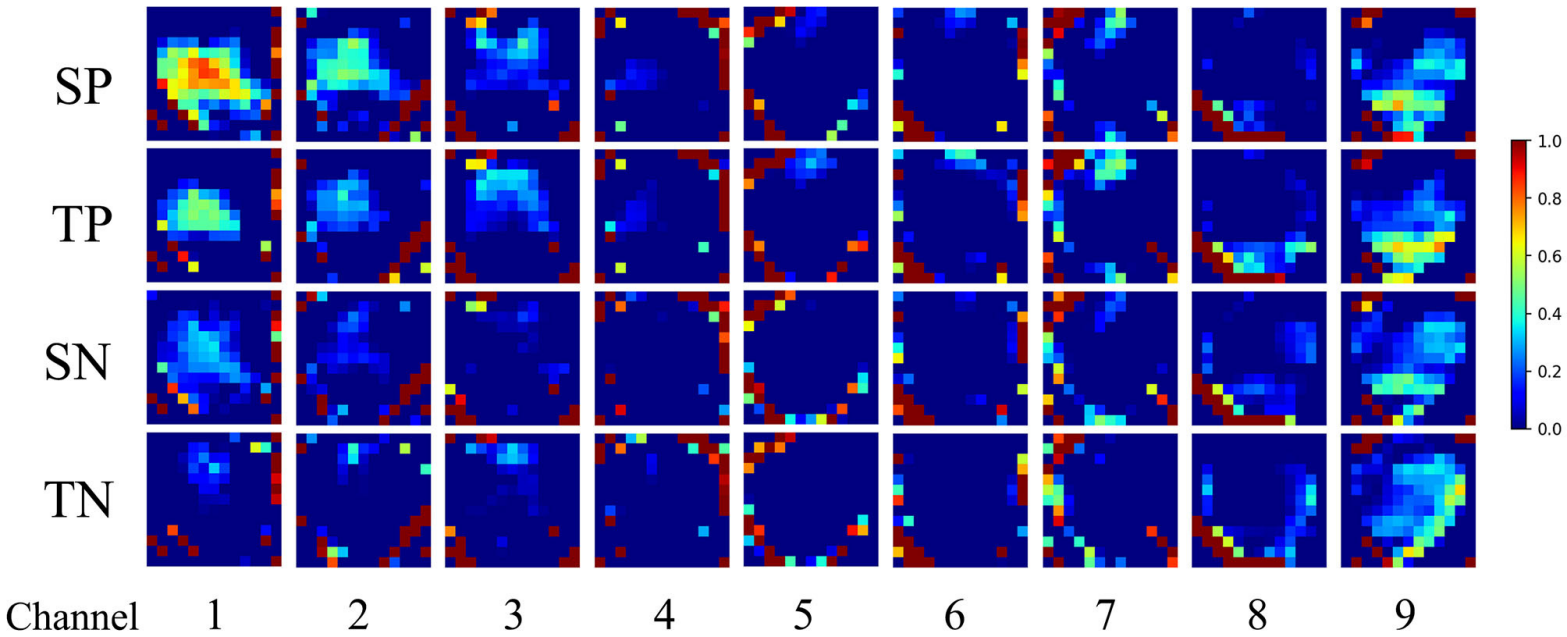
Feature visualization based on cross-day transfer model learning in the second convolutional layer. (SP, source positive; TP, target positive; SN, source negative; TN, target negative).

We studied the characteristics of the CNN learning EEG topological map. We extracted the output of the EEG topological map through the last layer of the convolutional network, and after superimposing and averaging the samples of the source and target domains, we selected nine channels with clear features and drawn feature maps after using maximum and minimum standardization, as shown in Figure 5. The first two rows represent the positive characteristics of the source domain and target domain learned by the convolutional network, and the last two rows represent the negative characteristics of the source domain and target domain learned by the network. From channels 1, 2, 3, and 4, we can see that there are differences between positive and negative emotions in the central area of the graph; in channels 5, 6, and 7, there are differences at the top of the graph; in channels 8, There are differences on both sides of the graph; channels 9 are differences at the bottom of the graph. There were obvious differences between positive and negative emotions in the parietal, frontal, and temporal lobes. This result was consistent with that of Zhuang et al. (2018). In addition, the positive and negative emotions of the source and target domains were similar, which proved that the network proposed in this paper can effectively solve the problem of cross-day transfer.

Next, we used the traditional support vector machine (SVM) classification method as the baseline, the RBF kernel is used, and compared the superior traditional transfer method, transfer component analysis (TCA), and the depth domain adaptation network DANN. First, we verified the EEG data set we collected using the leave-one-out method, and the results are shown in Table 3. In the self-built database, due to the difference in the data distribution of the training set and the test set, the baseline SVM classification performance was poor. In the second classification, for Joy-Sadness, Joy-Anger, and Joy-Fear, the accuracy rates were 70.02%, 71.16%, and 69.01%, and the accuracy rate for the four categories was 40.29%.Compared with the SVM method, the classification accuracy was slightly improved with the traditional TCA transfer method, but the improvement was not obvious. Using the DANN, the classification accuracy was significantly improved. The accuracy of the two classifications was 80.84%, 81.27%, and 80.20%, and the accuracy of the four classifications was 49.67%. Compared with the baseline SVM classifier, the accuracy of the classification was improved by 10%, 10%, 11%, and 9%. This showed that deep neural networks can effectively learn more transferable features for domain adaptation. The accuracy of the method proposed in this paper reached 84.0%, 87.04%, and 85.32% in the second classification. The accuracy of the four classifications reached 56.88%. Compared with the DANN network, it increased by 4%, 6%, 5%, and 7% respectively.

**Table 3:** Performance of adaptive methods in different domains for self- built EEG data set (cross-day).

**Methods**	**Two classification**						**Four classification**	
	**Joy-Sadness**		**Joy-Anger**		**Joy-Fear**			
	**Mean**	**Std**.	**Mean**	**Std**.	**Mean**	**Std**.	**Mean**	**Std**.
SVM	0.7002	0.159	0.7160	0.162	0.6901	0.137	0.4029	0.102
TCA	0.7429	0.172	0.7343	0.149	0.7256	0.131	0.4373	0.108
DANN	0.8084	**0.123**	0.8127	0.128	0.8020	**0.117**	0.4967	**0.083**
MMD	0.7997	0.153	0.8094	0.136	0.8038	0.118	0.4298	0.105
TDANN	**0.8400**	0.149	**0.8704**	**0.119**	**0.8532**	0.120	**0.5688**	0.097

Moreover, we used SEED data set for cross-day transfer research. The SEED data set was proposed by Zheng and Lu (2017). They used scores (1–5) and keywords to evaluate subjects' emotions (positive, neutral, and negative) when watching video clips. There were 15 movie clips (5 positive, 5 neutral, and 5 negative) and each movie clip lasted about 4 minutes. Fifteen healthy subjects (8 females, 7 males, MEAN: 23.27, SD: 2.37) were selected and scanned using the ESI NeuroScan System. The distribution of 62 electrodes conformed to the international 10–20 standard and the sampling rate was 1000 Hz. The EEG signal was down-sampled to 200 Hz, the signals that were heavily polluted by EOG and EMG were screened, and the screened signals were then passed through a 0.3–50 Hz bandpass filter. Then the EEG signal was divided into 1s-long data segments without overlap. Thus, there were 3,394 samples for each subject, and the sample sizes of the three emotions were basically the same. Each subject had three experiments. We used the leave-one-out method for cross-validation. The results are shown in the Table 4. Compared with SVM, TCA, DANN, and MMD, the accuracy of TDANN is improved by 16, 6, 5, and 6% respectively.

**Table 4:** Performance of SEED adaptation methods in different domains for the public data set (cross-day).

	**SVM**	**TCA**	**DANN**	**MMD**	**TDANN**
Mean	0.5884	0.6827	0.6972	0.6817	**0.7493**
Std.	0.1142	0.1670	**0.0900**	0.1350	0.0927

**Figure 6:**
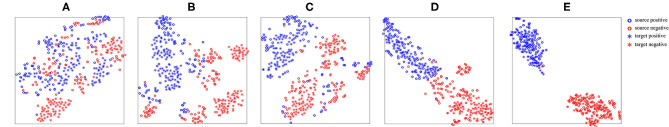
Feature visualization diagram. **(A)** original distribution of the features of the source and target domains; **(B)** distribution of the features after being mapped by the TCA algorithm; **(C)** distribution of the features learned by the DANN algorithm; **(D)** distribution of the features learned by the MMD algorithm; **(E)** feature distribution of TDANN learning.

In order to show the transfer process of feature distribution, we selected one subject's EEG data in our self-built data set to visualize by t-SNE (Donahue et al., 2013) in different domain adaptation algorithms in the leave-one-out method verification (see Figure 6). Figure 6A shows the original distribution of the source and target domains of the subject. It can be seen that the distribution of EEG features in the source and target domains was different, which was confusing and resulted in a very poor classification effect using the SVM classifier directly. Figure 6B shows the feature distribution map after feature mapping by the TCA method. It can be seen that mapping the feature to the feature subspace effectively distinguished the source domain from the target domain, but for multi-source domains transfer it was not enough; the feature distribution of the source domain was still very scattered. Figure 6C shows the feature distribution map learned by the DANN network. Still, some of the features of the source and target domains were confused, and the features of the source and target domains were relatively scattered and not clustered together. Figure 6D shows the distribution of features learned by the MMD. It can reduce the intra class distance, but can't widen the class spacing. Figure 6E shows the distribution of features learned by our method. It is evident that the features learned by our method are easier to distinguish than those learned by the DANN. Moreover, the class spacing became larger and the class inner distance became smaller.

### Cross-Subject Transfer Research

Currently, the most used data set for cross-subject transfer research is SEED, so we first chose to use SEED for this as well. When using the SEED data set to verify the cross-subject transfer research, we also used the leave-one-out method for cross-validation, that is, one subject was randomly selected as the test set, and the rest were the training set, so 15 iterations were required. Compared with the cross-day transfer study, the tasks were different, and the selected data and sample sizes were also different. The number of samples in the cross-day transfer study was small, while the number in the cross-subject transfer study was large. Therefore, the CNN in the cross-subject transfer study had a deeper network structure than in the cross-day transfer study. The CNN structure is shown in Table 5. Similarly, we added an AdaBN layer after each convolutional layer and fully connected layer. The AdaBN standardized the distribution between the source and target domains in each batch of samples, making the source and target domains better in the subspace matched by one (Donahue et al., 2013). In addition, each fully connected layer used a dropout layer, with a dropout rate of 0.5.

**Table 5:** CNN model structure for cross-subject transfer research.

**Layer**	**Input dimension**	**Output dimension**	**Kernel size**	**Stride size**
Conv1	5	32	3 × 3	1 × 1
Conv2	32	32	3 × 3	1 × 1
Maxpool1	32	32	2 × 2	2 × 2
Conv3	32	64	3 × 3	1 × 1
Conv4	64	64	3 × 3	1 × 1
Conv5	64	128	3 × 3	1 × 1
Conv6	128	128	3 × 3	1 × 1
Maxpool2	128	128	2 × 2	2 × 2
FC1	6 × 6 × 128	1,024		
FC2	1,024	512		
FC3	512	256		

We then conducted cross-subject transfer research on the SEED data set. When using the SEED data set to verify the cross-subject transfer research, we also used the leave-one-out method for cross-validation, that is, we randomly selected one subject as the test set, and the rest as the training set. Therefore, 15 iterations were required. The batch size was 224, the source domain and target domain were each 112, and the number of neurons in the fully connected layer was 1,024, 512, and 256, respectively. The hyperparameters were λ_*d*_ (0.1), λ_*m*_ (0.1), λ_*z*_ (0.01), and λ_*L*_ (0.1). An Adam optimizer was used, and the learning rate was 0.0005.

We simultaneously compared the current best-performing algorithms in the cross-subject transfer of EEG emotions, including shallow algorithms such as TCA and TPT, and deep algorithms such as DANN, DResNet, and WGAN-DA. We continued to use the SVM classifier as the baseline. Table 6 shows the average and variance obtained with different algorithms. Among the shallow transfer algorithms, TPT had the best effect, with an accuracy rate of 75.17%. Among the deep transfer algorithms, WGAN-DA had the best classification performance, with an accuracy rate of 87.07%. Although the accuracy of DResNet was not as high as that of WGAN-DA, DResNet did not use any information about the target domain data. TDANN's recognition accuracy rate was 87.9%, the highest recognition rate achieved by any of the algorithms, and it was more stable than WGAN-DA.

**Table 6:** Performance of SEED adaptation methods in different domains for the public data set (cross-subject).

	**SVM**	**TCA**	**TPT**	**DANN**	**MMD**	**DAN**	**DResNet**	**WGAN-DA**	**TDANN**
Mean	0.5818	0.6400	0.7517	0.7919	0.6655	0.8381	0.8530	0.8707	**0.8790**
Std.	0.1385	0.1466	0.1283	0.1314	**0.0483**	0.0856	0.0832	0.0714	0.0613

*In the mean, bolding means the accuracy mean is the largest; In the Std, the bold is the smallest*.

Then, we used a self-built data set for cross-subject transfer research. Twelve subjects' EEG data collected for the first time were used in this cross-subject transfer experiment. We used the leave-one-out method for cross-validation, and compared with TCA, DANN, and MMD algorithms. The results are shown in the Table 7. The accuracy of the method TDANN reached 83.79, 84.13, and 81.72% in the second classification. The accuracy of the four classifications reached 47.28%. Compared with the MMD, it increased by 5, 5, 6, and 4%, respectively. However, in the cross-subject transfer experiment of self-built data set, the overall accuracy is lower than that of cross day transfer experiment. The reason for this may be that there exists intrinsic differences among subjects, and more data collected from different subjects are needed to remove this intrinsic differences among subjects.

**Table 7:** Performance of adaptive methods in different domains for self-built EEG data set (cross-subject).

**Methods**	**Two classification**						**Four classification**	
	**Joy-Sadness**		**Joy-Anger**		**Joy-Fear**			
	**Mean**	**Std**.	**Mean**	**Std**.	**Mean**	**Std**.	**Mean**	**Std**.
SVM	0.6726	0.147	0.6995	0.1474	0.6565	0.120	0.3411	0.089
TCA	0.7505	**0.040**	0.7544	0.049	0.7327	0.459	0.4202	**0.025**
DANN	0.7299	0.046	0.7168	**0.023**	0.6624	**0.025**	0.4120	0.043
MMD	0.7837	0.151	0.7993	0.154	0.7568	0.146	0.4341	0.100
TDANN	**0.8379**	0.155	**0.8413**	0.137	**0.8172**	0.130	**0.4728**	0.079

*In the mean, bolding means the accuracy mean is the largest; In the Std, the bold is the smallest*.

## Conclusions

Emotion recognition is the most important part of human-computer interaction. EEG emotion recognition research has been developed for decades, and many impressive results have been obtained. However, there are still quite a few problems, among which the most important are cross-day transfer and cross-subject transfer. Because EEG signals are non-stationary, the signal distribution of each subject is different. Even for the same subject, there are differences in the EEG signals collected at different times.

In this paper, we propose a domain adaptation framework using deep neural networks for EEG emotion recognition. We have verified the performance of the framework on two data sets: our self-built data set, and the public data set SEED. In the cross-day transfer evaluation, we compared the currently favored transfer algorithms TCA and DANN. In the self-built data set, the accuracy rates of Joy-Sadness, Joy-Anger, and Joy-Fear were 84.0, 87.04, and 85.32%, respectively, and the accuracy rate of the four categories was 56.88%. In the SEED data set, the accuracy of three classification reached 74.93%. For the cross-subject transfer evaluation, the algorithm we proposed achieved an average accuracy rate of 87.9% in SEED data set. In the self-built data set, the accuracy rates of Joy-Sadness, Joy-Anger, and Joy-Fear were 83.79, 84.13, and 81.72%, respectively, and the accuracy rate of the four categories was 47.28%. Visualizing the features learned by the feature extractor, it can be clearly seen that different brain regions are activated by different emotions. The energy of positive emotions in the parietal, and frontal lobes is significantly higher than that of negative emotions.

In our cross-day transfer research, although we established a data set with the largest amount of data available at present for deep neural network training, the amount of data is still far from enough. The labor and funds required to build a sufficiently large data set are beyond the scope of most research institutions. Some studies have found that sample generation through a generative adversarial network (GAN) can effectively increase sample size and improve the training performance of a neural network to a certain extent. In follow-up research, we will study data enhancement based on a GAN to further address the problem of EEG emotion transfer.

## Data Availability Statement

The raw data supporting the conclusions of this article will be made available by the authors, without undue reservation.

## Ethics Statement

Written informed consent was obtained from the individual(s) for the publication of any potentially identifiable images or data included in this article.

## Author Contributions

GB is mainly responsible for research design, data analysis, and manuscript writing of this study. NZ is mainly responsible for data collection and data analysis. LT is mainly responsible for research design and data analysis. JS is mainly responsible for data collection and production of charts. LW is mainly responsible for data analysis and document retrieval. BY is mainly responsible for research design and manuscript writing. YZ is mainly responsible for data collection and manuscript writing. ZS is mainly responsible for data collection. All authors contributed to the article and approved the submitted version.

## Conflict of Interest

The authors declare that the research was conducted in the absence of any commercial or financial relationships that could be construed as a potential conflict of interest.
